# The impact of Extremely Low Frequency Electro- Magnetic Fields on Depression Anxiety and Stress

**DOI:** 10.1192/j.eurpsy.2024.783

**Published:** 2024-08-27

**Authors:** I. Kacem, I. Jammeli, A. Ghenim, A. Aloui, A. Chouchane, M. Bouhoula, A. Brahem, H. Kalboussi, O. El Maalel, S. Chatti, A. Kekeni, K. Bouzabia, M. Maoua, N. Mrizak

**Affiliations:** ^1^Occupational Medicine Department, Farhat Hached Academic hospital; ^2^Medical Department, Electricity and Gas Company, Sousse, Tunisia

## Abstract

**Introduction:**

According to World Health Organisation (WHO), Extremely Low Frequency Electro-Magnetic Fields (ELF-EMF) include frequencies ranging from 0 to 300 Hz. They are widespread in our daily life and in the workplace. These fields have an impact on physical and mental health including depression and anxiety.

**Objectives:**

To assess the impact of chronic occupational exposure to ELF-EMF on Depression, Anxiety and Stress among workers in the Tunisian Electricity and GasCompany of Sousse, Tunisia.

**Methods:**

In this cross-sectionalstudy, participants were enrolled into two groups: an “exposed group” including workers in a power plant and an “unexposed group” including administrative workers belonging to the same company. The Exposure to ELF EMFs was assessed by spot measurements using a portable magnetometer. Depression,Anxiety and Stress were assessed by the the Depression,Anxiety and Stress Scale(DASS-21).

**Results:**

This study included 77 exposed subjects and 88 unexposed subjects. The median age was 37 years for the exposed group and 43,5 years for the unexposed ones. Almost half of the exposed group were technicians and had a work experience of 9 years. The median value of EMF was 5,86 uT in the power plant[Min 0,1 Max 40,34 ut].The interpretation of DASS-21 showed that 24.7% of the exposed group and 3.4% of the unexposed group had depression (p<10-3). Anxiety was reported by, 23.4% of the exposed group and by none of the unexposed group. Stress was observed among 46.8% of the exposed group and by none of the unexposed group. After multivariate analysis, ELF-EMF exposure was significantly associated only with depression (p<10^-3^ ; OR=1,45 [1,17-1,81]) ).

**Conclusions:**

Chronic occupational exposure to ELF-EMF increases the risk ofDepression, anxiety and Stress. Underlying mechanisms are not established yet suggesting the need of further studies.

**Disclosure of Interest:**

None Declared

